# Workflow standardization of a novel team care model to improve chronic care: a quasi-experimental study

**DOI:** 10.1186/s12913-017-2240-1

**Published:** 2017-04-19

**Authors:** Laura Panattoni, Lily Hurlimann, Caroline Wilson, Meg Durbin, Ming Tai-Seale

**Affiliations:** 1 Hutchinson Institute for Cancer Outcomes Research, Fred Hutchinson Cancer Research Center, 1100 Fairview Avenue North, Seattle, WA 98109 USA; 2 600 Hermosa Way, Menlo Park, CA 94025 USA; 3 All Native Group, 9A Parkway Apt 201, Greenbelt, MD USA; 4 Canopy Health, 6475 Christie Avenue, Suite 360, Emeryville, CA 94608 USA; 5 Palo Alto Medical Foundation Research Institute, 2350 W El Camino Real, Mountain View, CA 94040 USA

**Keywords:** Standard work, Chronic care, Clinical outcomes

## Abstract

**Background:**

Team-based chronic care models have not been widely adopted in community settings, partly due to their varying effectiveness in randomized control trials, implementation challenges, and concerns about physician acceptance. The Palo Alto Medical Foundation designed and implemented “Champion,” a novel team-based model that includes new standard work (e.g. proactive patient outreach, pre-visit schedule grooming, depression screening, care planning, health coaching) to support patients’ self-management of hypertension and diabetes. We investigated whether Champion improved clinical outcomes.

**Methods:**

We conducted a quasi-experimental study comparing the Champion clinic-level intervention (*n* = 38 physicians) with a usual care clinic (*n* = 37 physicians) in Northern California. The primary outcomes, blood pressure and glycohemoglobin (A1c), were analyzed using a piecewise linear growth curve model for patients exposed to a Champion physician visit (*n* = 3156) or usual care visit (*n* = 8034) in the two years prior and one year post implementation. Secondary outcomes were provider experience, compared at baseline and 12 months in both the intervention and usual care clinics using multi-level ordered logistic modeling, and electronic health record based fidelity measures.

**Results:**

Compared to usual care, in the first 6 months after a Champion physician visit, diabetes patients aged 18-75 experienced an additional -1.13 mm Hg (95% CI: -2.23 to -0.04) decline in diastolic blood pressure and -0.47 (95% CI: -0.61 to -0.33) decline in A1c. There were no additional improvements in blood pressure or A1c 6 to 12 months post physician visit. At 12 months, Champion physicians reported improved experience with managing chronic care patients in 6 of 7 survey items (*p* < 0.05), but compared to usual, this difference was only statistically significant for one item (*p* < 0.05). Fidelity to standard work was uneven; depression screening was the most commonly documented element (85% of patients), while care plans were the least (30.8% of patients).

**Conclusions:**

Champion standard work improved glycemic control over the first 6 months and physicians’ experience with managing chronic care; changes in blood pressure were not clinically meaningful. Our results suggest the need to understand the relationship between the intervention, the contextual features of implementation, and fidelity to further improve chronic disease outcomes. This study was retrospectively registered with the ISRCTN Registry on March 15, 2017 (ISRCTN11341906).

**Electronic supplementary material:**

The online version of this article (doi:10.1186/s12913-017-2240-1) contains supplementary material, which is available to authorized users.

## Background

The customary chronic care management delivered by a single primary care physician leads to gaps between evidence-based best practices and the status quo [1, 2]. In a typical short appointment, physicians may offer preventative services, manage multiple chronic conditions, educate patients and families, and provide numerous other services. Patient self-management and lifestyle change can greatly alter the course of chronic conditions, but physicians may lack the time, training or financial incentives to use evidence-based techniques that foster patient self-efficacy [3]. The responsibility of managing these complex patients contributes to physician job dissatisfaction [4] and burnout [5]. Randomized controlled trials (RCT) of team-based models that employ nurse care managers to provide patient self-management improve glycohemoglobin (A1c), low-density lipoprotein, and mood in patients with depression [6], along with diabetes and/or heart disease [7]. Other models use unlicensed health coaches to perform some of a nurse care manager’s tasks, such as patient education, participatory problem solving, and action planning [1, 2, 8].

Despite substantial previous efforts [2, 6–9], widespread adoption of team-based chronic care remains slow for several reasons. There is limited published information about the clinical effectiveness of translating these RCTs into real world practice. Implementation is challenging because the new care elements are more complex and wide ranging than those required for episodic or preventive care, they may challenge well-established practice habits, and the number of chronic care patients vary across practices and day to day within a given practice [10]. Provider fidelity to new standard work can be hard to measure when it involves communication practices such as motivational interviewing and action planning. Pragmatically evaluating the effectiveness of a new care model in a real world community setting is difficult, partly because patients may have varying degrees of exposure or none at all [11].

The Palo Alto Medical Foundation developed “Champion,” a novel team care model that includes new standard work for patients with hypertension and diabetes. Champion redesigned primary care visits, expanding the medical assistant’s pre-visit role to gather more information and take measurements, while the physician offered evidence-based patient self-management support, and unlicensed health coaches worked with selected patients between physician visits. The implementation involved a two day multifaceted training with extensive live practice, fidelity checks and three review workshops. The primary aim of this study was to determine whether the Champion standard work improved disease control. The secondary aim was to report the physician’s experience with delivering chronic care and the Champion standard work, fidelity to the standard work, and general utilization metrics.

## Methods

### Study design and setting

**Fig. 1:**
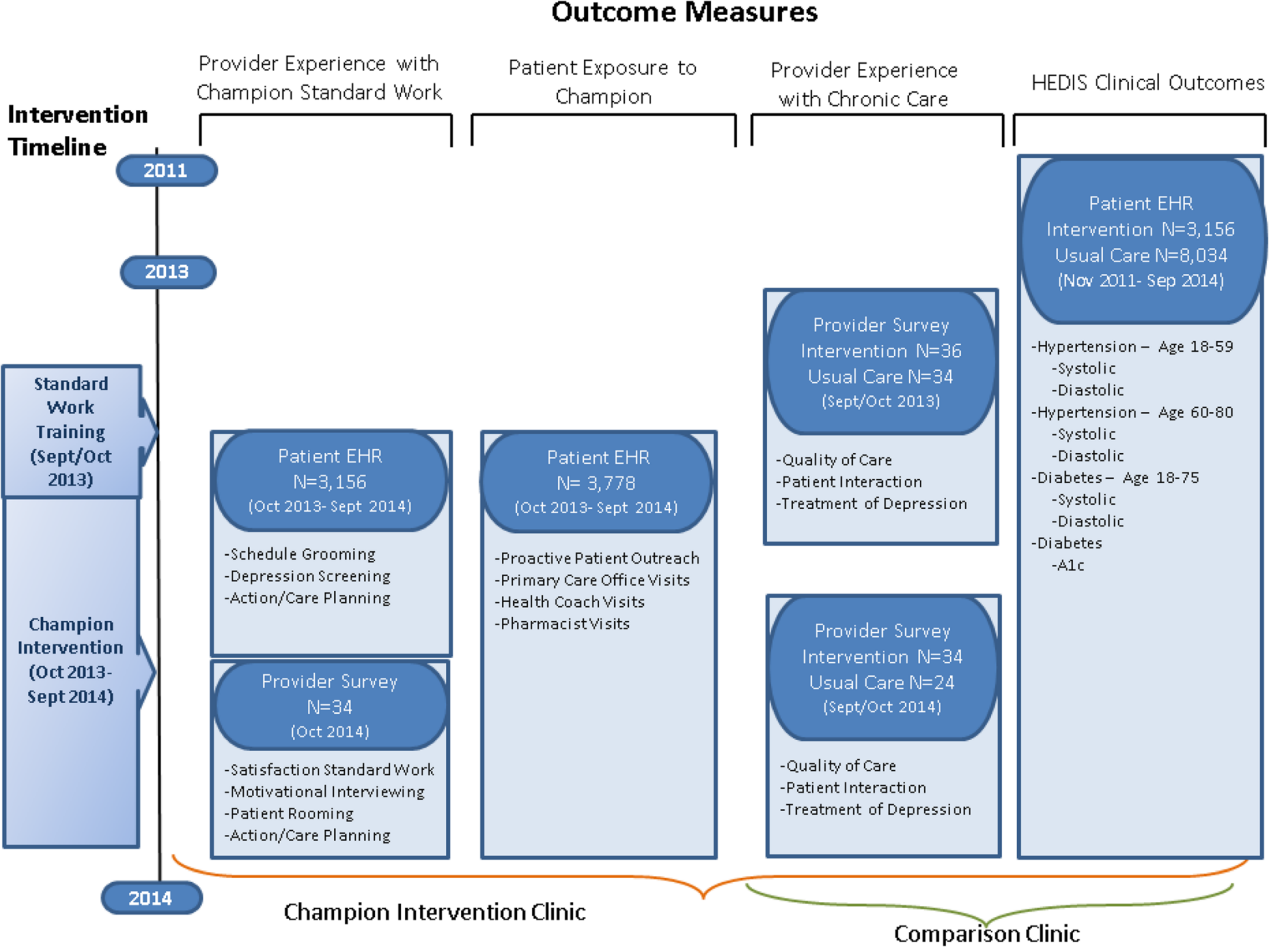
Flow diagram of the study. EHR; Electronic health record

This quasi-experimental study was conducted in two primary care clinics in a non-profit multispecialty group practice, with approximately 450 primary care physicians who were paid fee-for-service (Fig. 1). The insurance mix for the medical group was 67% fee-for-service, 15% capitated, 13% Medicare, and 5% Medicaid and other. The Champion model was implemented in the family and internal medicine departments of one clinic with 38 physicians serving approximately 12,000 adult patients with diabetes or hypertension. The intervention clinic was chosen by the medical group’s administrators. The comparison clinic, with 37 physicians serving approximately 17,000 patients with these diagnoses, was chosen by the study investigators because it had the closest number of physicians and chronic care patients to the intervention clinic.

### Intervention

#### Overview of the champion care pathway

The Champion care team included a physician, an unlicensed medical assistant, a nurse care manager, two unlicensed health coaches, and a pharmacist. Unlike in previous RCTs which targeted patients with poor biometric control [2, 6–9], Champion included all patients because glycemic control and blood pressure can fluctuate over time. The first step in the Champion care pathway was proactive patient outreach, where health coaches reached out to patients who were in poor biometric control (blood pressure ≥ 140/90, A1c ≥ 7.5), had not seen their physician or endocrinologist in the last three months, and had no scheduled appointments in the upcoming month. Each week, physicians prioritized a list of eligible patients and health coaches scheduled appointments, calling up to two times by telephone and then sending secure messages via the electronic health record (EHR). Approximately two thirds of the patients with poor biometric control were eligible for outreach.

The goal of schedule grooming was to address the care gap that many patients did not complete required labs or bring home monitoring logs and medication lists to their visits. Two weeks before a planned office visit, the physician and the medical assistant “groomed” the schedule to determine each patient’s requirements (e.g. lab tests, home monitoring logs) and the medical assistant contacted the patients.

The Champion physician visit incorporated features from several evidence-based chronic care models [2, 6–9]. Similar to the Teamlet model [1, 2], Champion expanded the medical assistant’s role to collect more patient data and perform some basic exams during the rooming process. Medical assistants entered data into Champion text fields (“Smartphrases”) in the EHR, screened patients for depression using the Patient Health Questionaire (PHQ-9) and performed diabetic foot exams [12]. Unlike previous models [2, 6–9], in which nurse care managers or unlicensed providers conducted patient education, participatory problem solving, and action planning, Champion intended to free up time in the office visit for the physicians to provide these services and then engage the care team for ongoing care when necessary. Physician’s new standard work included motivational interviewing [13], a brief evidence-based therapeutic method to encourage behavior change, and completing a care plan with the patient.

For additional post-visit care, Physicians referred patients to two new resources: a health coaching program and/or a pharmacist. The health coaching program blended previous chronic care models [2, 6–9] and included 2 non-licensed health coaches overseen by a nurse care practitioner. The health coaches employed motivational interviewing to provide ongoing self-management support; while the nurse care manager mainly conducted the tasks requiring licensure (e.g., medication adjustments). The pharmacist provided medication reconciliation.

#### Training providers in the champion standard work

In September 2013, the physicians, medical assistants, nurse care manager, and health coaches received two days of training in the Champion standard work (the pharmacist only participated when relevant). The training was designed according to evidence-based best practices for educational outreach to providers [10, 14]. A detailed description and agenda for the two day training is available as Additional file 1. The execution of the training workshop and the development of the training materials was completed by clinic physician leaders, internal lean management consultants, and managers (including the vice-president of operations).

For each component of the standard work, the training consisted of multimedia instruction with supporting materials, live real time practice, and tailored debriefing and coaching for each physician-medical assistant team. The multimedia presentation included power point slides, an instructional video, and large flip charts on the walls with the training objectives for each component. Providers were given a standard workbook with detailed instructions and links to a library of instructional videos, which were referenced during the presentation. The live real time practice was completed with each provider’s patients and the standard work documentation, including the 50 Champion EHR text fields.

The live practice involved different exercises to reinforce each component of the standard work. For patient outreach, physicians listed enough patients from their own panels for the first month. For schedule grooming, the physician-medical assistant teams reviewed their upcoming chronic care appointments, determined what the patients needed to complete or bring, and contacted the patient. The office visit standard work included a practice session of motivational interviewing for physicians and the rooming process for medical assistants. Physicians were given hypothetical patients to refer to the health coaching program and the pharmacist. The physician-medical assistant teams also returned to their offices and completed a Champion office visit with one of their own chronic care patients. In addition to the two day training, the health coaches received 40 h of training related to chronic disease care, goal setting, documentation, identifying barriers, and professional boundaries, and 20 h of in-depth motivational interviewing instruction [15].

After the provider training, the clinic’s managers transitioned into assessing provider fidelity to the workflow. For each physician-medical assistant team, they conducted three quarterly chart reviews and one or two “process walks” where they observed the team complete tasks, including motivational interviewing, against a checklist. Providers who were observed to have room for improvement were engaged in a conversation to address barriers, review standard work, and create memory aids. Clinic’s managers also conducted two hour review workshops with all the providers 30, 60, and 90 days post launch of Champion.

### Outcomes

#### Clinical outcomes of patients with physician office visits

We compared the clinical outcomes of patients who had a Champion physician visit versus patients seen in usual care visits in a comparison clinic between Oct 1, 2011 and Sept 30, 2014. Patients in the study were age 18 years or older, had no severe mental illness, dementia, or substance abuse disorders, and had a designated primary care physician in the family or internal medicine departments of either clinic at some point during the three-year period. All patients had either a diagnosis of diabetes (ICD 9 codes 249.x, 250.x) or hypertension (ICD 9 code 401.x) on their problem list. Eligible patients also had a visit to their designated physician where an encounter diagnosis of diabetes or hypertension was recorded during the observation period. Finally, all patients were required to have a relevant clinical outcome measurement in the EHR (for all, blood pressure; and for only diabetes: A1c) on or before the date of the indexed physician office visit.

Clinical outcomes for diabetes and hypertension were determined from the Healthcare Effectiveness Data and Information Set (HEDIS) guidelines [16]. For hypertension, these included blood pressure < 140/90 mm Hg for ages 18-59 and blood pressure < 150/90 mm Hg for ages 60-80. For diabetes, these included blood pressure < 140/90 mm Hg for ages 18-75, and A1c > 9.0.

#### Physician experience with chronic care

For the secondary aim, we assessed the impact of the Champion standard work on physician’s experience with chronic care at baseline (before the training) and at 12 months using a survey in both the intervention and usual care clinic. All questions explicitly referred to patients with hypertension and/or diabetes. We evaluated quality of care and patient interaction using items from the American Medical Group Association (AMGA) Provider Satisfaction Survey but altering the questions to address only patients with diabetes and/or hypertension [17]. The AMGA is a validated tool to assess physician experience in a scale from 1 (Very Dissatisfied) to 4 (Very Satisfied). We also assessed physician’s perceived time for and attitudes towards the treatment of depression using two peer reviewed items with a scale that ranges from 1 (Strongly Disagree) to 4 (Strongly Agree) [18].

#### Physician experience and fidelity to the champion standard work

One year post implementation, we surveyed physicians about their experience with the Champion care pathway via a questionnaire. We evaluated physician’s satisfaction with the standard work for patients with hypertension and diabetes separately using a scale from 1 (Very Dissatisfied) to 4 (Very Satisfied). Physicians reported how confident they were in their ability to use motivational interviewing on a scale from 0 (Not Confident at All) to 10 (Completely Confident). Physicians were asked if Champion standard work reduced the time they spent gathering information from patients during visits on a scale from 1 (Strongly Disagree) to 4 (Strongly Agree). Physicians also checked all the ways they conduct care planning from a set of four options: discuss goals informally, use the paper care plan, use the electronic care plan, other.

To measure fidelity to the Champion standard work for office visits, we reported the percentage of patients with the following documented standard work in the EHR over the first year of the intervention: schedule grooming, depression screening (PHQ2 or PHQ9), and an electronic care plan. The denominator was the number of patients with a Champion physician office visit (Table 5).

#### Patient exposure to the champion care pathway

To determine the number of patients in the intervention clinic who experienced Champion standard work, we analyzed the use of Champion related text fields in the EHR between Oct 2013 and September 2014. We reported five utilization measures, the number of patients who experienced i) at least one documented element of Champion standard work, ii) patient outreach and subsequently had a Champion physician office visit, iii) a Champion physician office visit, iv) a health coach visit, and v) a pharmacist visit.

### Statistical analyses

#### Primary outcomes

To investigate whether Champion improved clinical outcomes, we used a piecewise linear growth curve model (linear mixed model with splines) to test the differential change in clinical outcomes over time, between patients in Champion and the usual care clinic, before and after a physician visit. We used an exposure, instead of an intention to treat, approach because only 27% of the patients on the Champion clinic’s active patient list with hypertension or diabetes had a physician office visit during the one-year observation period (see Table 5). Because each patient’s first Champion physician visit might occur at any time during the one-year observation period, we compared clinical outcomes among patients with the same length of exposure to the intervention by giving the first office visit an index date of 0 and comparing outcomes with the same index date post visit. For example, if a patient had her first office visit on January 1^st^ and a lab test for A1c on April 1^st^, the lab would have an index day of 89 days post visit.

Our analyses accounted for patient’s sex, self-reported race/ethnicity, age, insurance type, and Charlson Comorbidity Score [19] at the patient-year level. We identified the patient’s designated physician at each visit date and included the physician’s sex, specialty, and number of providers in department. Summary statistics of the patient panel characteristics based on sex, age, race/ethnicity, insurance type, Charlson Comorbidity Score [19], panel size, and diagnoses of hypertension, diabetes, or depression were included at the physician-year level. An indicator variable accounted for patients with more than one physician visit during the intervention time period. We addressed missing data in the predictor variables by including a category for unknown whenever possible.

To account for the possibility of patient self-selection into the Champion versus usual care clinic, we used propensity score weighting based on patient characteristics and six additional utilization metrics (number of office visits to primary care, endocrinology, nephrology, cardiology, and all specialists along with the number of telephone calls) in the period before the first office visit [20–22]. A propensity score model was estimated with a separate logistic regression for each of the four cohorts (e.g. Hypertension: Age 18-59). A pre-determined standardized mean difference of 0.1 was used to determine adequate balance between patients in the Champion versus usual care clinic in each of the four cohorts [23]. After propensity score weighting, all controls were well balanced with standardized mean differences less than 0.1 in each of the four cohorts. Additionally, after propensity score weighting, control variable mean values were qualitatively similar (Additional file 2).

We used a piecewise linear growth curve model [24] to compare how the outcomes of the two clinics changed over time. Compared to interrupted time series [25], growth curve modeling is well suited to datasets of patients who have a different number of outcome measurements at varying frequencies, and has been used in other recent evaluations of health interventions [26]. Because RCTs of primary care interventions often include outcome measurements at six months and one year, we used the piecewise linear model to account for differences in outcomes during the first 180 days and the second 180 days after each patient’s indexed physician office visit. The model was specified as:

$$ \begin{array}{l}\mathbb{E}\left[ Outcom{e}_{i t}\right]={\theta}_0+{\theta}_1\  Intervention\  Clini{c}_i+{\theta}_2\  Before\kern0.5em  First\  Office\  Visi{t}_t\\ {}\kern10em +{\theta}_{3\ }1-180\  Days\  Post\  Visi{t}_t\\ {}\kern10em +{\theta}_4181-365\  Days\  Post\  Visi{t}_t+{\theta}_5 Intervention\  Clini{c}_i X\  Before\  Visi{t}_t\\ {}\kern10em +{\theta}_6 Intervention\  Clini{c}_i X\ 1-180\  Days\  Post\  Visi{t}_t\\ {}\kern10em +{\theta}_7 Intervention\  Clini{c}_i X\ 181 - 365\  Days\  Post\  Visi{t}_t+{X}_{i t}^{\hbox{'}}\beta \end{array} $$

in which *i* indicated a patient and *t* represented an indexed day. $\mathbb{E}\left[ Outcom{e}_{it}\right]$ denoted the mean HEDIS clinical measurement and *X* denoted patient characteristics. The coefficient *θ*_1_ measured the mean difference in the outcome of the intervention clinic compared to the usual care clinic at the same indexed date. The coefficients *θ*_2_, *θ*_3_, and *θ*_4_ measured the slope (mean increase in outcome per half year) of the usual care clinic during the referenced index time period. The coefficients *θ*_5_, *θ*_6_, and *θ*_7_measured the difference in the slope of the intervention clinic versus the usual care clinic during the referenced index time period. The model also included provider and actual (not indexed) quarter fixed effects with an unstructured covariance matrix. All analyses were performed using Stata/MP 13.0 XTMIXED. The unit of analysis was at the patient-day level but the results were reported semi-annually.

#### Secondary outcomes

For the secondary aim, we assessed the impact of Champion standard work on physicians’ experience with chronic care. We measured their beliefs in the domains of Quality of Care, Patient Interaction, Patient Influence, and Treatment of Depression at baseline (before the training) and 12 months post launch in both the intervention and usual care clinic. We measured the post minus pre difference in each clinic separately. We also used a difference in differences methodology to compare the size of the difference between the two clinics. For these analyses, we estimated multilevel ordered logit models at the physician level, to account for the clustering of time periods within physicians.

### Ethical approval

The Palo Alto Medical Foundation Institutional Review Board approved the human subject protection protocol. Written informed consent for data collection and usage were obtained from all providers included in the study. De-identified data collection from the EHR was approved by the institutional review board.

## Results

### Clinical outcomes of patients with primary care office visits

The analysis of the clinical outcome included 3,156 patients with a Champion physician visit and 8,034 patients with a usual care visit during the time period between Oct 1, 2011 and Sept 30, 2014. The percentage of hypertension patients in the Champion vs. usual care clinic was 21.0% (2,488/11,873) vs. 42.9% (7,320/17,049), while the percentage of patients with diabetes was 14.5% (1,718/11,873) vs. 17.3% (2,941/17,049) respectively (Table 1). The patient populations of the two clinics were different demographically in terms of gender, age, race, ethnicity, and insurance (*p* < 0.001), but not in terms of their Charlson comorbidity index score (*p* = 0.37) [19].

**Table 1:** Patient and provider characteristics at the primary care office visit

	Champion Intervention Clinic Patients (*n* = 3156)		Usual Care Clinic Patients (*n* = 8034)		*P* Value
Problem List diagnoses (*n* = 11,190)					
Hypertension Only, # (%)	1,257	39.83	4,413	54.93	<0.001
Diabetes Only, # (%)	609	19.30	573	7.13	
Hypertension & Diabetes, # (%)	894	28.33	1,870	23.28	
Hypertension & Depression, # (%)	181	5.74	680	8.46	
Diabetes & Depression, # (%)	59	1.87	141	1.76	
Hypertension & Diabetes & Depression, # (%)	156	4.94	357	4.44	
Patient characteristics (*n* = 11,190)					
Female, # (%)	1,403	44.46	4,002	49.81	<0.001
Age, years, mean (SD)	56.82	0.22	61.89	0.15	<0.001
Race/Ethnicity, # (%)					
White	685	21.70	3,802	47.32	<0.001
Asian	1,176	37.26	2,507	31.20	
Hispanic	259	8.21	720	8.96	
Other	677	21.45	907	11.29	
Unknown	359	11.38	98	1.22	
Insurance, # (%)					
PPO	1,845	58.46	3,673	45.72	<0.001
HMO	580	18.38	992	12.35	
Medicaid	12	0.38	112	1.39	
Medicare FFS	613	19.42	2,484	30.92	
Medicare HMO	86	2.72	714	8.89	
Self	19	0.60	59	0.73	
Other	1	0.03	0	0.00	
Charlson Comorbidity Score, mean (SD)	1.21	0.02	1.18	0.02	0.37
Physician characteristics (*n* = 75)					
Female, # (%)	27	71.05	24	64.86	0.57
Specialty, # (%)					
Family Medicine	23	60.53	14	37.84	<0.001
Internal Medicine	15	39.47	23	62.16	
Patient panel characteristics (*n* = 75)					
% Female, # (%)	55.54	3.96	55.47	3.53	0.99
Age, yrs,, mean (SD)					
% 18 ≤ Age < 35	28.38	1.48	23.87	2.27	0.10
% 35 ≤ Age < 50	38.39	1.38	30.89	1.36	<0.001
% 50 ≤ Age < 65	23.67	1.38	25.18	1.34	0.40
% Age ≥ 65	9.56	0.83	20.06	2.38	<0.001
Race/Ethnicity, mean (SD)					
% White	18.79	1.64	45.82	2.34	<0.001
% Asian	38.05	1.80	31.62	2.45	0.04
% Hispanic	6.58	0.54	7.86	0.78	0.18
% Other	21.88	0.90	11.27	0.54	<0.001
% Unknown	14.70	0.50	3.43	0.28	<0.001
Insurance, mean (SD)					
% PPO	59.43	0.79	53.90	1.64	<0.001
% HMO	14.31	0.48	11.97	0.41	<0.001
% Medicaid	0.26	0.03	0.67	0.05	<0.001
% Medicare FFS	6.88	0.56	12.57	1.43	<0.001
% Medicare HMO	0.87	0.13	3.82	0.71	<0.001
% Self	1.12	0.06	1.41	0.06	<0.01
% Other	17.13	1.04	15.66	1.38	0.40
Mean Charlson Comorbidity Score, mean (SD)	0.34	0.02	0.45	0.04	<0.001
% Hypertension, mean (SD)	17.17	0.90	26.85	3.72	0.02
% Diabetes, mean (SD)	7.12	0.41	8.89	1.46	0.25
% Depression, mean (SD)	7.78	0.44	11.70	1.06	<0.001
Mean Panel Size, mean (SD)	1,536.82	88.02	1,800.35	126.96	0.09

Chi-square or t-tests were used as appropriate. *P* value <0.05 was considered statistically significant. *PPO* Preferred Provider Organization, *HMO* Health Maintenance Organization, *FFS* Fee for service

Table 1 displayed the summary statistics of the changes in clinical outcomes for the two clinics over three time periods, i.e., before the office visit, and the first and second 6 month period post visit. The same patients in the period before the office visit were tracked over time, however, at least one third of the patients did not have a blood pressure or glycemic testing within 6 months of their physician visit, and at least two thirds did not have a measurement after 6 months. At the time of the first office visit, 27.2% (860/3,156) of patients had at least one measurement above the HEDIS threshold in the Champion clinic compared to 29.1% (2,338/8,034) in usual care. Out of the 3,156 Champion patients, 85.0% had a depression assessment and 400 (14.9%) of those “tested positive” for possible depression (PHQ2 score ≥3 or PHQ9 score ≥5) [12]. In contrast, <1% of comparable patients in the usual care clinic were screened for depression.

**Fig. 2:**
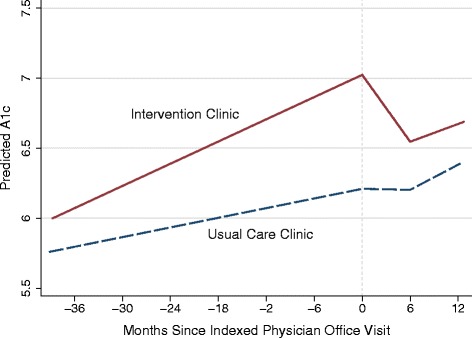
Predicted A1c for a hypothetical patient who is non-Hispanic white, male, aged 50, with PPO insurance. A1c: glycohemoglobin; PPO: Preferred provider organization; PCP: Primary care physician

We compared the difference in clinical outcomes between patients in the intervention and usual care clinics, before and after the index office visit. The unadjusted difference-in-difference growth curve modeling results (Table 2) indicated that the statistically significant outcomes for blood pressure were either not sustained or not clinically meaningful in the fully adjusted results (Table 3). Compared to patients in the usual care clinic, Champion patients age 18 – 75 with diabetes experienced an additional decrease of -1.13 mm Hg (95% CI: -2.23 to -0.04) for diastolic blood pressure over the 6 month period. However, these results were not clinically meaningful and were not sustained in the following 6 through 12 months post the index visit. There were no other results for systolic or diastolic blood pressure for any cohort in either period. However, among Champion patients with diabetes, statistically significant decline was noted early on (-0.47 points on average after six months, 95% CI: -0.61 to -0.33) but not in the subsequent six to twelve months (Fig. 2).

**Table 2:** Summary statistics of HEDIS clinical outcomes for champion and usual care patients with a primary care office visit

	Champion Intervention Clinic (*n* = 3156)			Usual Care Clinic (*n* = 8034)			Unadjusted *p* value*	
	Before First Office Visit	1-180 Days Post Visit	181-365 Days Post Visit	Before First Office Visit	1-180 Days Post Visit	181-365 Days Post Visit	Intervention Clinic X 1-180 Days After Visit	Intervention Clinic X 181-365 Days After Visit
Hypertension - Age 18-59 (*n* = 4,385)								
Systolic, mean (SD)	1,31.5 (12.4)	129.3 (14.4)	129.3 (15.7)	134.3 (13.3)	132.3 (14.3)	132.5 (15.5)	0.11	0.05
Diastolic, mean (SD)	82.3 (7.9)	79.7 (9.2)	79.8 (9.2)	83.7 (8.5)	82.0 (9.3)	81.8 (9.7)	0.07	0.12
Num patients per period	1,449	762	303	2,936	1,667	781		
Hypertension - Age 60-80 (*n* = 4,620)								
Systolic, mean (SD)	133.3 (12.1)	131.3 (14.3)	130.8 (16.4)	134.2 (13.1)	134.7 (15.7)	133.6 (15.4)	<0.001	0.01
Diastolic, mean (SD)	75.6 (7.9)	73.1 (8.7)	72.9 (8.5)	75.7 (8.0)	74.7 (9.3)	74.4 (9.4)	<0.01	0.55
Num patients per period	983	652	335	3,637	2,356	1,325		
Diabetes- Age 18-75 (*n* = 3,768)								
Systolic, mean (SD)	126.5 (12.7)	126.1 (14.6)	125.5 (15.3)	129.8 (13.2)	130.4 (15.2)	129.8 (15.7)	<0.01	0.16
Diastolic, mean (SD)	76.4 (7.7)	74.5 (8.8)	74.4 (8.8)	76.2 (7.9)	75.6 (9.4)	74.9 (9.4)	<0.001	0.22
Num patients per period	1,565	942	478	2,203	1,474	950		
Diabetes (*n* = 4,442)								
A1c, mean(SD)	7.5 (1.4)	7.5 (1.3)	7.4 (1.5)	7.3 (1.3)	7.4 (1.3)	7.4 (1.3)	<0.001	0.13
Num patients	1,688	829	447	2,754	1,595	1,035		

All dates are indexed to the date of the first physician office visit during the intervention period from 10/2013-9/2014. Before First Office Visit includes all dates up to and including the date of the first physician office visit. The same patients in the period Before First Office Visit are tracked over time, however, not all have clinical measurements in the two post visit period. The two interaction terms Intervention Clinic x 1-180 Days Post Visit and Intervention Clinic x 181-365 Days Post Visit measure the difference in the slop of the intervention clinic compared to the usual care clinic during the indexed time period. *P value <0.05 was considered statistically significant. *P* values refer to results of a piecewise linear growth curve model including only a constant, plus the clinic and time period variables (e.g. variables included in Table 5). A1c: glycohemoglobin

**Table 3:** Adjusted results from a piecewise linear growth curve model of HEDIS clinical outcomes for patients with a primary care office visit

	Hypertension: Age18-59		Hypertension: Age 60-80		Diabetes: Age 18-75		Diabetes
	Systolic	Diastolic	Systolic	Diastolic	Systolic	Diastolic	A1c
	Est. (95% CI)	Est. (95% CI)	Est. (95% CI)	Est. (95% CI)	Est. (95% CI)	Est. (95% CI)	Est. (95% CI)
Intervention Clinic	0.71 (-4.25,5.67)	-0.96 (-4.87,2.94)	-1.18 (-6.05,3.69)	-1.5 (-3.84,0.83)	0.47 (-4.27,5.22)	-2.4 (-5.08,0.28)	0.81 (0.42,1.21)^b^
Before First Office Visit	0.33 (-0.34,1.00)	0.35 (-0.08,0.78)	0.31 (-0.41,1.04)	-0.26 (-0.65,0.14)	0.62 (-0.24,1.47)	0.23 (-0.26,0.72)	0.07 (-0.00,0.14)
1-180 Days Post Visit	-2.44 (-3.80,-1.09)^b^	-1.57 (-2.37,-0.76)^b^	-1.12 (-2.35,0.11)	-0.85 (-1.51,-0.19)^a^	-0.66 (-2.01,0.69)	-0.27 (-1.02,0.48)	-0.01 (-0.12,0.11)
181-365 Days Post Visit	3.44 (0.72,6.16)^a^	2.15 (0.53,3.78)^b^	0.62 (-1.64,2.88)	0.04 (-1.17,1.25)	3.21 (0.66,5.76)^a^	1.24 (-0.10,2.58)	0.18 (-0.00,0.36)
Intervention Clinic x Before Visit	-0.28 (-0.67,0.11)	-0.35 (-0.60,-0.09)^b^	-0.21 (-0.62,0.20)	-0.13 (-0.35,0.09)	0.05 (-0.34,0.44)	-0.15 (-0.37,0.07)	0.09 (0.05,0.13)^b^
Intervention Clinic x 1-180 Days Post Visit	-0.75 (-2.82,1.31)	-0.58 (-1.87,0.71)	-0.96 (-2.86,0.95)	-1.03 (-2.07,0.01)	-1.65 (-3.68,0.39)	-1.13 (-2.23,-0.04)^a^	-0.47 (-0.61,-0.33)^b^
Intervention Clinic x 181-360 Days Post Visit	1.7 (-3.95,7.35)	0.86 (-2.34,4.05)	4.31 (-0.70,9.33)	0.52 (-2.06,3.10)	2.88 (-2.30,8.06)	0.37 (-2.64,3.38)	-0.05 (-0.39,0.29)
Observations	39,181	39,170	57,592	57,580	45,787	45,778	23,339
Num Patients	4,385	4,385	4,620	4,620	3,768	3,768	4,442

^a^Statistically significant at the 5% probability level, ^b^Statistically significant at the 1% probability level. The above variables specify the piecewise linear model. The analysis included a constant, patient level, provider level, and patient-panel level control variables, provider and actual quarter fixed effects, and an unstructured covariance matrix. The indicator for Usual Care Clinic was left out. Results are reported at the half yearly level. Interpretation of the variables:

Intervention Clinic measures the mean difference in the outcome of the intervention clinic compared to the usual clinic at the same index time. Before Office Visit, 1-180 Days Post Visit, 181-365 Days Post Visit measures slope (mean increase in outcome per quarter) of the usual care clinic during the indexed time period. Intervention Clinic x Before Office Visit, Intervention Clinic x 1-180 Days Post Visit, Intervention Clinic x 181-365 Days Post Visit measures the difference in the slop of the intervention clinic compared to the usual care clinic during the indexed time period. Est: Estimate; 95% CI: 95% Confidence Interval

Subgroup analyses of patients above each HEDIS threshold before their visit found no statistically significant results for blood pressure but A1c outcomes trending towards statistical significance (Additional file 3). However, additional analyses of diabetes patients with A1c outcomes above thresholds similar to the previously mentioned RCTs (e.g. A1c ≥8.5, A1c ≥8.0) found statistically significant results [7, 8]. Among patients with diabetes and a pre-visit A1c greater than or equal to 8.5, Champion patients tested in the first six months experienced an additional A1c decrease of -0.71 (95% CI: -1.21 to -0.21). For patients with a pre-visit A1c greater than or equal to 8.0, the additional decrease was -0.75 (-1.14, -0.36). These results were not sustained in the subsequent six months (Additional file 4).

### Physician experience with chronic care

Thirty-six physicians (response rate = 94.7%) in the Champion clinic participated in the baseline survey (Table 4). At 12 months, they reported statistically significant improvements for all of the questions except for the item “diagnosing and treating depression is my responsibility”. Pertaining to patients with diabetes and/or hypertension, this study found a significant increase in physician satisfaction with the quality of care they were able to provide (baseline vs. 12 months: 3.03 vs. 3.46, *p* < 0.01) and the expectations/demands of their patients (baseline vs. 12 months: 2.73 vs. 3.20, *p* < 0.01). Physicians were also less likely to report that patients with chronic conditions have so many problems that they didn’t always have time to consider depression (baseline vs. 12 months: 3.12 vs. 2.34, *p* < 0.001).

**Table 4:** Provider’s experience with chronic care at baseline and 12 months and experience with Champion standard work

		Champion Intervention Clinic				Usual Care Clinic					
Experience with Chronic Care^a^	Scale range	Baseline (*N* = 36)	12 months (*N* = 34)	Diff	*p* value	Baseline (*N* = 34)	12 months (*N* = 24)	Diff	*p* value	DID	*p* value
Quality of Care											
Quality of care you are able to provide	(1-4)	3.03	3.46	0.43	<0.01	3.12	3.52	0.40	0.04	0.03	0.98
Patient Interaction											
Your relationships with patients	(1-4)	3.06	3.43	0.37	0.01	3.54	3.69	0.15	0.26	0.97	0.32
Expectations/demands of your patients	(1-4)	2.73	3.20	0.47	<0.01	3.12	3.32	0.20	0.16	1.27	0.17
Adherence/compliance with instructions of your patients	(1-4)	2.58	2.94	0.36	0.01	2.85	3.07	0.22	0.10	1.01	0.36
Continuity of patient care you are able to provide	(1-4)	3.03	3.41	0.38	0.01	3.31	3.55	0.24	0.13	0.66	0.42
Treatment of Depression											
Patients with chronic conditions have so many problems that I don’t always have time to consider depression	(1-4)	3.12	2.34	-0.78	<0.001	2.78	2.48	-0.30	0.10	-1.80	0.03
Diagnosing and treating depression is my responsibility	(1-4)	3.21	3.29	0.08	0.38	3.26	3.28	0.02	0.99	0.53	0.59
Experience with Champion Standard Work											
Satisfaction with standard work for hypertension	(1-4)		3.03								
Satisfaction with standard work for diabetes	(1-4)		3.17								
Confidence in ability to use motivational interviewing	(0-10)		6.48								
Champion medical assistant rooming procedures have reduced the time I spend on gathering information during our visits	(1-4)		2.67								
How do you do care planning? (Check all that apply)											
Discuss goals informally	% *N* = 34		62.50								
Discuss goals with a paper care plan	% *N* = 34		7.50								
Discuss goals with an electronic care plan	% *N* = 34		52.50								
Response Rate		94.7%	85.0%			91.9%	61.5%				
Total Number of Providers		38	40			37	39				

^a^All questions referred to patients with hypertension and/or diabetes. Multilevel ordered logit models accounting for cluster of time periods within patients were used, *p* value <0.05 was considered statistically significant. Quality of Care and Patient Interaction come from the American Medical Group Provider Satisfaction Survey and Treatment of Depression comes from an Attitudes Towards Treatment and Diagnosis of Depression Survey. *Diff* Difference between 12 months and baseline, *DID* Difference in Differences of survey items = difference between 12 months and baseline for the intervention clinic - difference between 12 months and baseline for the comparison clinic

In the usual care clinic, 34 physicians (response rate = 91.7%) participated in the survey at baseline and 24 (response rate = 61.5%) at 12 months. There was a marginal improvement at 12 months for two of the questions; physician satisfaction with the quality of care they were able to provide (baseline vs. 12 months: 3.12 vs. 3.52, *p* = 0.04) and how often they felt they positively influenced the lives of patients through their work (baseline vs. 12 months: 4.33 vs. 5.15, *p* = 0.04). However, the difference-in-difference results were only statistically significant (*p* < 0.05) for one item. Compared to usual care physicians, Champion physicians reported a greater improvement in their experience that patients with chronic conditions have so many problems that they didn’t always have time to consider depression (Champion difference vs. usual care difference: -0.78 vs. -0.30, *p* < 0.03).

### Physician experience and fidelity to the champion standard work

Thirty-four physicians (response rate = 85%) in the Champion clinic participated in the survey 12 months following the training workshop (Table 4). Physicians were on average “Somewhat satisfied” (mean scores 3.03 and 3.17) with their standard work for patients with hypertension and diabetes, respectively. They also indicated an above average level of confidence in their ability to use motivational interviewing (mean score 6.48). However, they were split (mean score 2.67) on whether the rooming procedures reduced their time spent gathering information during the visit. Finally, the most common method of action planning reported was informal discussion (62.5% of physicians), followed by use of the electronic care plan (52.5%), while the least commonly used method was the paper care plan (7.5%).

As measured by documentation in the Champion EHR text fields, fidelity to Champion’s office visit standard work was uneven across components (Table 5). Out of the 3156 patients with a Champion physician visit, the most commonly documented component was depression screening (85% of patients) while the least documented was the electronic care plan (30.8% of patients). Schedule grooming was found in 32.9% of the patient charts before the visit.

**Table 5:** Patient exposure to the champion care pathway

Patient Outreach	
Num Patients Contacted	984
Num (%) Patients with a Resulting Primary Care Provider Visit	323 (32.8%)
Primary Care Provider Visit	
Num Patients with a Primary Care Provider Visit	3,156
Num (%) Patients with Depression Screen	2,683 (85.0%)
Num (%) Patients with Schedule Grooming	1,038 (32.9%)
Num (%) Patients with Care Plan (.CCMCAREPLAN)	972 (30.8%)
Extended Care Team Visits	
Num Patients with a Health Coach Visit	169
Num Patients with a Pharmacist Visit	176
Total Num Patients on the Champion Clinic’s Active Patient List with Hypertension or Diabetes	11,873

### Patient exposure to the champion care pathway

There were 11,873 patients on the Champion clinic’s active patient list with hypertension or diabetes (Table 5). For patient outreach, the 2 health coaches reached out to 984 patients, which was 64.4% (984/1529) of the eligible population. Among those, 32.8% completed a Champion physician office visit. Out of the 3156 patients who had a Champion physician visit, 74.8% (2,362/3156) had only one Champion physician visit during the year. After Champion physician visits, 169 patients saw a health coach and 176 patients saw the pharmacist. During the intervention period from Oct 2013 to September 2014, 26.6% (3,156/11,873) of the eligible patients in the intervention clinic had a Champion physician visit compared to 47.1% (8034/17,049) of the eligible patients in the usual care clinic who had a primary care visit with an encounter diagnosis of diabetes or hypertension.

## Discussion

This study examined whether the development and implementation of a standard workflow that redesigned primary care visits to enhance the self-management support provided by physicians and established a health coaching program, improved disease control for patients with hypertension and diabetes. Compared to usual care, the clinical outcomes associated with Champion physician visits included a six month decline of -0.47 (95% CI: -0.61 to -0.33) in A1c for patients with diabetes and a clinically marginal short term decline in blood pressure among some patients with hypertension or diabetes. Subgroup analysis indicated that among diabetes patients with A1c ≥ 8.5, the Champion cohort experienced a six month decline of -0.71 (95% CI: -1.21 to -0.21).

The clinical outcomes in this study compared favorably to those found in other recent trials of team-based chronic care (which only included patients in poor biometric control) [7, 8, 10]. Previous RCTs in hypertension showed no significant changes in systolic blood pressure at one year [7, 8, 10]. For patients with diabetes, a RCT of visits every two to three weeks with a nurse care manager demonstrated a decline in A1c of -0.56 (95% CI: -0.85 to -0.27) at one year [7], while a safety net clinic with medical assistants providing health coaching every three months had a higher proportion of patients with diabetes meeting a goal of 8.0 [8]. Of the patients with at least one physician visit in this study, approximately 23% had poor biometric control (A1c ≥ 8.5), and they visited their physician on average every three to four months.

Champion physicians reported some improvements in their experience delivering chronic care in the areas of the quality of care, patient interaction, and time for the treatment of depression. A goal of Champion was to provide more time for the physician to offer self-management support and other care. Physicians reported they had more time to consider depression, which may have come from additional time freed up by the medical assistants’ rooming practices.

Analysis of the Champion EHR text fields and the 12 month physician survey indicated the standard work was not always delivered as designed. Less than one third of patients with a Champion physician office visit had a documented care plan or evidence of pre-visit schedule grooming. Fidelity to the standard work may have been uneven for numerous reasons including 1) greater detail and complexity within Champion encounters (e.g. 50 Champion EHR text fields); 2) varying numbers of patients eligible for the Champion intervention across physician practices and from day to day in a given practice; 3) challenges engaging physicians and other team members to change well-established practice habits; and 4) physicians reported they were on average “somewhat satisfied” with the Champion standard work. Measuring fidelity by the documented use of Champion EHR text fields has limitations. For example, many physicians reported they preferred to conduct their care planning on an informal basis. However, over 85% of patients with a Champion office visit had a documented depression screen, compared to <1% in usual care. At 12 months post intervention, physicians reported they were “fairly confident” in their use of motivational interviewing, which provided some evidence of their willingness to adopt new practice habits [27]. Future research could qualitatively explore any implementation barriers that may have resulted in the low uptake of some components of the standard work.

The intervention clinic’s eligible patients had relatively limited exposure to the Champion standard work. The uptake of the health coaching program and the pharmacist was low indicating that Champion functioned mostly as a physician-medical assistant rather than an extended provider team model. Recruitment and uptake issues for the health coaching program were explored in another paper [15]. Further research could explore what the acceptability and uptake of the previously described nurse care manager [7] and medical assistant health coaching models [8] might be in different settings. From a population management perspective, Champion had no effect on the clinical outcomes of the clinic’s total population of active patients with these diagnoses because only 27% of these patients had a Champion physician visit and the active patient turnover (gaining and losing eligible patients over the study’s timeframe) was approximately 9%. Future research could address whether the clinic’s definition of the active patient population (any service billing in the last two years) was too inclusive or whether there could be greater uptake of Champion physician visits. Champion did improve outreach to patients in poor biometric control, who were not keeping up with the physician visits, which resulted in an additional 323 visits.

There are several limitations arising from the observational nature of the study. Use of a comparison group along with propensity score stratification cannot rule out the possibility that unmeasured factors, not Champion workflow, caused the observed outcomes. Also, at least one third of the patients did not have a blood pressure or glycemic testing within 6 months of their physician visit, and at least two thirds did not have measurements in the subsequent 6 months. It is possible that the statistical methods did not adequately control for differences between patients with and without post visit labs. Some patients had their Champion physician visit at the end of the evaluation period introducing censorship bias. Lastly, these results are from one large multispecialty delivery organization, whose patients are mostly privately insured, potentially limiting generalizability to other settings.

## Conclusions

The Champion standard work, along with the training and fidelity monitoring, produced some promising clinical results in diabetes management, depression screening and positive chronic care experiences for physicians. The findings in this study suggest that improvements in A1c found in recent team-based chronic care RCTs can be replicated in real world clinical practice, despite the different providers (physician, nurse care manager, unlicensed health coach) conducting the patient self-management support. This supports the generalizability of evidence-based self-management support (e.g. motivational interviewing) across provider types and settings. However, the standard work had mixed results in terms of hypertension management, physician experience with the standard work, and patient exposure. One open question is which provider type is most cost-effective to provide the self-management support. Our experience is that additional visits with other team members, such as health coaches, might have low uptake. Clinical effectiveness of Champion might be strengthened by exploring why A1c outcomes failed to improve six months after the initial physician visit and why some standard work components (e.g. pre-visit schedule grooming and care planning) had low fidelity.

Workflow standardization is an important tool for incorporating evidence into routine care but must also be adaptable and responsive to on-going quality improvement. This is critical as the number of chronic conditions managed through Champion or similar workflows expands. Champion was a significant organizational investment in chronic care, which the delivery organization has viewed as an initial step in a long process of continuous quality improvement [28]. Its long term sustainability should be evaluated as the organization moves further into the era of accountable care where value of services brings more reward than volume.

## Additional files

Additional file 1: Champion Chronic Care Management Training. This file includes a two day schedule for the two day Champion providers’ standard workflow training. (PDF 206 kb)

Additional file 2: Propensity Score Weighting Metrics. This file includes the area under the ROC curve and the standardized mean differences of the characteristics of the patients in the Intervention vs Usual Care Clinic, for the four cohorts. (PDF 424 kb)

Additional file 3: Clinical Outcomes for Patients with Clinical Outcomes above the HEDIS Threshold at the time of the Office Visit. This file includes the results of Tables 3, 4 and 5 for those patients with poor biometric control at the time of the primary care provider office visit. (PDF 819 kb)

Additional file 4: Adjusted A1c Outcomes for Diabetes Patients with Pre-visit A1c ≥ 9.0, A1c ≥ 8.5, and A1c ≥ 8.0. This file includes the results of Table 5 for diabetes patients with Pre-visit A1c ≥ 9.0, A1c ≥ 8.5, and A1c ≥ 8.0. (PDF 313 kb)

## Abbreviations

A1c: Glycohemoglobin
AMGA: American Medical Group Association
DID: Difference in differences
Diff: Difference between 12 months and baseline
EHR: Electronic health record
FFS: Fee for service
HMO: Health Maintenance Organization
PPO: Preferred Provider Organization
RCT: Randomized control trial
