# Supplementary material for: Workflow standardization of a novel team care model to improve chronic care: a quasi-experimental study
Source: BMC Health Serv Res. 2017 Apr 19;17:286. doi: 10.1186/s12913-017-2240-1 (PMC5395876; doi:10.1186/s12913-017-2240-1)

Champion | Chronic Care Management Training

DAY 1: Pre-Visit

TIME	PROVIDERS	FLOW MANAGERS
8:00	Kickoff	
9:00	Break	
9:15	Clinical Guidelines	
9:50	Panel Management	
10:45	Preparing for CCM Visits: Overview	
11:30	Lunch	
12:00	Lunch, continued.	**Schedule Grooming**<br>**Weekly Huddle**<br>**Patient Prep Contact**
12:30	Thinking Differently about Preparing for Visits	
1:40	**Weekly Huddle**	
2:40	**Patient Prep Contact**<br>Live Practice	
3:00	Break	
3:15	Building the Team	
4:40	Experiment Expectations	
4:50	Process Check (+/-)	
5:00	Adjourn	

DAY 2: Visit

TIME	PROVIDERS	FLOW MANAGERS
8:00	Kick Off and Introduction	
8:45	Motivational Interviewing<br>Break<br>Overview of MA STW<br>**Prepare Visit Plan**<br>**Warm Handoff**<br>**Visit**<br>**Post Visit Follow Up**	Day 1 STW Follow-Up<br>**Rooming**<br>Break<br>**Warm Handoff**<br>**Post Visit Follow Up**
12:10	Lunch	
12:40	**Warm Handoff**<br>**Discharge**<br>**Daily Huddle**	
1:00	LIVE PRACTICE with Patients<br>Daily Huddle through Discharge	
3:45	Debrief & Coaching	
4:15	Continuous Improvement Process	
4:45	Process Check (+/-)	
5:00	Adjourn	

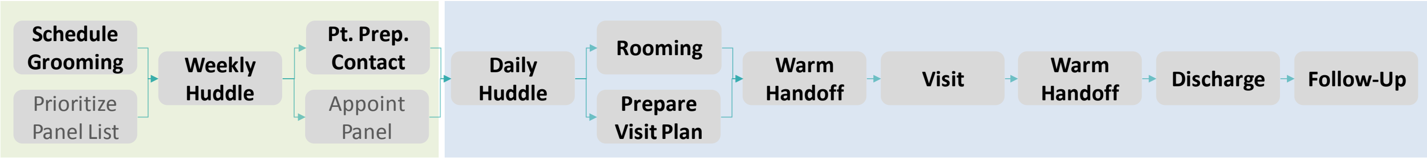

Supplement: Supplementary file 1 — Champion Chronic Care Management Training. This file includes a two day schedule for the two day Champion providers’ standard workflow training. (PDF 206 kb) [file 12913_2017_2240_MOESM1_ESM.pdf]
