# Supplementary material for: Workflow standardization of a novel team care model to improve chronic care: a quasi-experimental study
Source: BMC Health Serv Res. 2017 Apr 19;17:286. doi: 10.1186/s12913-017-2240-1 (PMC5395876; doi:10.1186/s12913-017-2240-1)
Supplement: Supplementary file 2 — Propensity Score Weighting Metrics. This file includes the area under the ROC curve and the standardized mean differences of the characteristics of the patients in the Intervention vs Usual Care Clinic, for the four cohorts. (PDF 424 kb) [file 12913_2017_2240_MOESM2_ESM.pdf]

Additional File 2: Propensity Score Weighting Metrics

The area under the ROC curve and the standardized mean differences for the four cohorts are provided below.

I. Hypertension: Age 18-59

Logistic model for the Propensity Score

number of observations = 4385
area under ROC curve = 0.7026

Additional File Table 1: Patient characteristics of the cohorts in the Intervention vs Usual Care Clinic, before the first office visit, with propensity score weighting

	Intervention Clinic Mean	Usual Care Clinic Mean	p-value for diff.	Standardized diff.
prop_score	0.33	0.33	0.88	0.006
Age	50.33	50.16	0.498	0.024
Female	0.44	0.43	0.685	0.014
PtAsian	0.35	0.35	0.986	-0.001
PtHispanic	0.09	0.09	0.967	-0.001
PtOther	0.16	0.16	0.981	-0.001
PtUnknown	0.05	0.05	0.879	0.005
Ins_HMO	0.2	0.2	0.765	0.01
Ins_Medicaid	0.02	0.01	0.837	0.012
Ins_MedicareFFS	0.02	0.02	0.908	0.004
Ins_MedicareHMO	0	0	0.9	0.007
Ins_Oth	0	0	.	.
PtCharlson	0.68	0.67	0.76	0.011
Num_OV primary_care	3.58	3.56	0.85	0.006
Num _OV_endocrinology	0.18	0.17	0.873	0.006
Num _OV_nephrology	0.05	0.07	0.33	-0.026
Num _OV_cardiology	0.16	0.16	0.971	0.001
Num _OV_allspecialist	3.89	3.82	0.727	0.01
Num _telephone_call	7.2	7.15	0.882	0.005

II. Hypertension: Age 60-80

Logistic model for the Propensity Score

number of observations = 4620
area under ROC curve = 0.7141

Additional File Table 2: Patient characteristics of the cohorts in the Intervention vs Usual Care Clinic, before the first office visit, with propensity score weighting

	Intervention Clinic Mean	Usual Care Clinic Mean	p-value for diff.	Standardized diff.
prop_score	0.22	0.21	0.66	0.018
Age	69.43	69.84	0.135	-0.069
Female	0.53	0.54	0.556	-0.026
PtAsian	0.27	0.27	0.939	0.003
PtHispanic	0.08	0.08	0.97	0.001
PtOther	0.14	0.14	0.798	0.008
PtUnknown	0.03	0.03	0.706	0.009
Ins_HMO	0.08	0.08	0.83	-0.007
Ins_Medicaid	0.02	0.01	0.587	0.128
Ins_MedicareFFS	0.5	0.52	0.529	-0.028
Ins_MedicareHMO	0.12	0.13	0.673	-0.022
Ins_Oth	0	0	.	.
PtCharlson	1.29	1.25	0.501	0.027
Num_OV primary_care	4.87	4.92	0.766	-0.011
Num _OV_endocrinology	0.27	0.27	0.947	0.002
Num _OV_nephrology	0.23	0.25	0.759	-0.012
Num _OV_cardiology	0.49	0.48	0.876	0.006
Num _OV_allspecialist	7.94	8.07	0.73	-0.013
Num _telephone_call	11.95	12.08	0.839	-0.009

III. Diabetes: Age 18-75

Logistic model for the Propensity Score

number of observations = 3767
area under ROC curve = 0.7062

Additional File Table 3: Patient characteristics of the cohorts in the Intervention vs Usual Care Clinic, before the first office visit, with propensity score weighting

	Intervention Clinic Mean	Usual Care Clinic Mean	p-value for diff.	Standardized diff.
prop_score	0.41	0.41	0.613	-0.027
Age	57.61	57.34	0.537	0.024
Female	0.45	0.45	0.852	0.007
PtAsian	0.38	0.39	0.703	-0.014
PtHispanic	0.11	0.11	0.888	-0.005
PtOther	0.16	0.16	0.96	-0.002
PtUnknown	0.05	0.05	0.727	-0.018
Ins_HMO	0.18	0.18	0.955	-0.002
Ins_Medicaid	0.02	0.01	0.887	0.008
Ins_MedicareFFS	0.23	0.22	0.628	0.019
Ins_MedicareHMO	0.05	0.05	0.705	0.023
Ins_Oth	0	0	0.317	0.034
PtCharlson	1.96	1.91	0.37	0.043
Num_OV primary_care	4.78	4.71	0.673	0.018
Num _OV_endocrinology	0.6	0.58	0.816	0.01
Num _OV_nephrology	0.24	0.24	0.962	0.002
Num _OV_cardiology	0.39	0.36	0.482	0.028
Num _OV_allspecialist	7.19	6.94	0.52	0.025
Num _telephone_call	12.47	11.74	0.295	0.05

IV. Diabetes: A1c

Logistic model for the Propensity Score

number of observations = 4441
area under ROC curve = 0.7348

Additional File Table 4: Patient characteristics of the cohorts in the Intervention vs Usual Care Clinic, before the first office visit, with propensity score weighting

	Intervention Clinic Mean	Usual Care Clinic Mean	p-value for diff.	Standardized diff.
prop_score	0.37	0.38	0.648	-0.023
Age	61.94	61.84	0.867	0.007
Female	0.48	0.47	0.751	0.012
PtAsian	0.36	0.38	0.529	-0.022
PtHispanic	0.11	0.11	0.897	0.005
PtOther	0.15	0.15	0.943	-0.002
PtUnknown	0.05	0.05	0.705	-0.018
Ins_HMO	0.15	0.15	0.897	-0.004
Ins_Medicaid	0.01	0.01	0.887	0.008
Ins_MedicareFFS	0.3	0.3	0.957	0.002
Ins_MedicareHMO	0.1	0.09	0.638	0.028
Ins_Oth	0	0	0.317	0.027
PtCharlson	2.21	2.14	0.333	0.05
Num_OV primary_care	5.43	5.35	0.635	0.02
Num _OV_endocrinology	0.63	0.6	0.663	0.019
Num _OV_nephrology	0.34	0.38	0.73	-0.016
Num _OV_cardiology	0.55	0.5	0.425	0.03
Num _OV_allspecialist	8.58	8.23	0.401	0.032
Num _telephone_call	14.74	13.71	0.239	0.063
