# Supplementary material for: Workflow standardization of a novel team care model to improve chronic care: a quasi-experimental study
Source: BMC Health Serv Res. 2017 Apr 19;17:286. doi: 10.1186/s12913-017-2240-1 (PMC5395876; doi:10.1186/s12913-017-2240-1)
Supplement: Supplementary file 3 — Clinical Outcomes for Patients with Clinical Outcomes above the HEDIS Threshold at the time of the Office Visit. This file includes the results of Tables 3, 4 and 5 for those patients with poor biometric control at the time of the primary care provider office visit. (PDF 819 kb) [file 12913_2017_2240_MOESM3_ESM.pdf]

Additional File 3: Clinical Outcomes for Patients with Clinical Outcomes above the HEDIS Threshold at the time of the Office Visit.

Table 3. Out of Control Patient and Provider Characteristics at the Primary Care Office Visit

	**Intervention Clinic (n= 732)**		**Usual Care Clinic (n= 2,737)**		
	Mean/#	***SD/%***	***Mean/#***	***SD/%***	***P Value***
Problem List diagnoses (n = 3,469)					
Hypertension Only	318	43.44	1588	58.02	<0.001
Diabetes Only	91	12.43	131	4.79	
Hypertension & Diabetes	236	32.24	646	23.60	
Hypertension & Depression	33	4.51	227	8.29	
Diabetes & Depression	10	1.37	20	0.73	
Hypertension & Diabetes & Depression	44	6.01	125	4.57	
Patient characteristics (n=3,469)					
Female	335	45.77	1341	49.00	0.13
Age, yrs	55.54	11.86	58.2	12.59	<0.001
Race/Ethnicity					
White	145	19.81	1222	44.65	<0.001
Asian	262	35.79	832	30.40	
Hispanic	57	7.79	275	10.05	
Other	180	24.59	360	13.15	
Unknown	88	12.02	48	1.75	
Insurance					
PPO	452	61.75	1461	53.38	<0.001
HMO	136	18.58	382	13.96	
Medicaid	2	0.27	45	1.64	
MedicareFFS	116	15.85	635	23.20	
MedicareHMO	20	2.73	182	6.65	
Self	6	0.82	32	1.17	
Other	0	0.00	0	0.00	
Charlson Score	1.15	1.27	1.06	1.47	<0.001
Physician characteristics (n=75)					
Female	27	71.1	24	64.9	0.74
Specialty					
Family Medicine	23	60.5	14	37.8	0.05
Internal Medicine	15	39.5	23	62.2	
Patient panel characteristics (n=75)					
% Female	55.5	4	55.5	3.5	0.99
Age, yrs					
% Age1	28.4	1.5	23.9	2.3	0.1

% Age2	38.4	1.4	30.9	1.4	<0.001
% Age3	21.6	1.4	23.2	1.3	0.4
% Age4	9.6	0.8	20.1	2.4	<0.001
Race/Ethnicity					
% White	18.8	1.6	45.8	2.3	<0.001
% Asian	38.1	1.8	31.6	2.5	0.04
% Hispanic	6.6	0.5	7.9	0.8	0.18
% Other	21.9	0.9	11.3	0.5	<0.001
% Unknown	14.7	0.5	3.4	0.3	<0.001
Insurance					
% PPO	59.4	0.8	53.9	1.6	<0.01
% HMO	14.3	0.5	12	0.4	<0.001
% Medicaid	0.3	0	0.7	0.1	<0.001
% MedicareFFS	6.9	0.6	12.6	1.4	<0.001
% MedicareHMO	0.9	0.1	3.8	0.7	<0.001
% Self	1.1	0.1	1.4	0.1	0.003
% Other	17.1	1	15.7	1.4	0.4
Mean Charlson Score	0.3	0	0.5	0	0.01
% Hypertension**	17.7	0.9	26.5	3.7	<0.01
% Diabetes**	7.3	0.4	8.8	1.5	0.35
% Depression**	8	0.4	11.7	1.1	<0.01
Mean Panel Size	1537	88	1800	127	0.09

* P value based on a ttest with unequal variances.
** Based on a diagnosis of diabetes (ICD 9 codes 249.%, 250.%), hypertension (ICD 9 code 401.%), or depression (ICD 9 codes 300.4, 311, 648.44, 296.%) on the problem list.
** Intervention Period 10/2013 – 9/2014

Thanks to Gabe' suggestions, we found some mistakes in my previous table, and the things I modified here include the counts under Problem List diagnoses and p-values for count data among groups(I used "Pearson's Chi-squared Test for Count Data" instead of "Test of Equal or Given Proportions"), and also the pvalue for Charlson Score since Gabe found the Charlson Score is not normally distributed, so I used "Mann-Whitney U Test" instead of "two sample t-test".

Table 4. Summary Statistics for Hypertension and Diabetes HEDIS Clinical Outcomes – Out of Control Patients

	**Intervention Clinic (n=732)**			**Usual Care Clinic (n=2,737)**			**P Values**	
	Pre Period 720-0 Days Before First Office Visit	***Post Period 1-180 Days After First Visit***	***Post Period 181-365 Days After First Visit***	***Pre Period 720-0 Days Before First Office Visit***	***Post Period 1-180 Days After First Visit***	***Post Period 181-365 Days After First Visit***	***Difference in Differences 1-180 Days After Visit***	***Difference in Differences 181-365 Days After Visit***
Hypertension - Age 18-59 (n=1,680)								
Systolic, mean (SD)	139.9 (11.6)	135.7 (14.9)	138.1 (15.9)	142.6 (12.4)	138.1 (14.0)	138.5 (16.0)	0.83	0.15
Diastolic, mean (SD)	87.7 (7.7)	84.3 (9.2)	84 (8.8)	88.6 (8.2)	85.6 (9.2)	85.2 (9.9)	0.51	0.43
Num patients per period	367	209	68	1313	778	346		
Hypertension - Age 60-80 (n= 1,074)								
Systolic, mean (SD)	143.8 (12.7)	141.9 (15.1)	141.1 (14.6)	146.3 (12.9)	144.3 (16.0)	142.6 (15.2)	0.59	0.15
Diastolic, mean (SD)	80.5 (8.8)	77.5 (9.7)	75.8 (7.1)	81 (8.7)	78.6 (9.9)	77.7 (9.3)	0.955	0.45
Num patients per period	148	101	43	926	623	322		
Diabetes- Age 18-75 (n=870)								
Systolic, mean (SD)	139.4 (10.8)	137.5 (14.3)	135.5 (14.8)	141.4 (13.0)	140.3 (15.0)	139.1 (16.5)	0.253	0.12
Diastolic, mean (SD)	82 (8.4)	78.5 (8.8)	78.1 (8.8)	80.5 (9.0)	79.2 (10.5)	78.2 (9.9)	0.247	0.74
Num patients per period	260	183	86	610	413	246		
Diabetes (n=472)								
A1c, mean(SD)	9.9 (1.7)	9.0 (1.7)	8.5 (2.0)	9.9 (1.5)	9.4 (1.9)	9.2 (1.8)	<0.01	0.7
Num patients	158	84	46	314	164	110		

Table 5. Adjusted Results from a Piecewise Linear Growth Curve Model of HEDIS Clinical Outcomes for Patients with a Primary Care Office Visit – Out of Control Patients

	**Hypertension: Age18-59**		**Hypertension: Age 60-80**		**Diabetes: Age 18-75**		**Diabetes**
	Systolic Est. (95% CI)	***Diastolic*** Est. (95% CI)	***Systolic*** Est. (95% CI)	***Diastolic*** Est. (95% CI)	***Systolic*** Est. (95% CI)	***Diastolic*** Est. (95% CI)	***A1c*** Est. (95% CI)
Intervention Clinic	1.85 (-12.86,16.57)	2.88 (-4.12,9.88)	-38.82 (-98.07,20.42)	1.54 (-26.02,29.10)	-1.26 (-8.01,5.48)	4.59 (-2.00,11.17)	-4.6 (-14.80,5.61)
Before First Office Visit	2.06 (0.94,3.17)**	1.5 (0.76,2.24)**	3.77 (1.92,5.61)**	1.03 (-0.03,2.09)	3.7 (1.85,5.55)**	0.83 (-0.36,2.02)	0.63 (0.35,0.91)**
1-180 Days Post Visit	-11.89 (-13.93,-9.84)**	-6.37 (-7.70,-5.04)**	-11.57 (-14.53,-8.60)**	-6.87 (-8.54,-5.21)**	-9.81 (-12.62,-6.99)**	-4.24 (-5.82,-2.67)**	-1.83 (-2.27,-1.38)**
181-365 Days Post Visit	4.37 (0.05,8.70)*	2.89 (0.20,5.58)*	2.59 (-1.84,7.01)	1.27 (-1.25,3.79)	1.27 (-3.91,6.46)	0.42 (-2.12,2.96)	1 (0.17,1.84)*
Intervention Clinic x Before Visit	-0.2 (-1.06,0.66)	-0.17 (-0.69,0.36)	0.61 (-0.35,1.57)	0.07 (-0.55,0.70)	0.83 (-0.13,1.79)	-0.05 (-0.61,0.51)	0.07 (-0.16,0.30)
Intervention Clinic x 1-180 Days Post Visit	0.01 (-4.69,4.71)	-1.31 (-3.74,1.11)	-2.85 (-6.97,1.27)	-1.15 (-5.14,2.84)	-2.5 (-6.31,1.31)	-0.71 (-3.35,1.94)	-0.66 (-1.33,0.003)
Intervention Clinic x 181-360 Days Post Visit	1.28 (-8.03,10.59)	2.95 (-3.59,9.50)	7.31 (-11.36,25.99)	-1.46 (-13.46,10.55)	3.73 (-7.93,15.40)	-3.12 (-10.56,4.32)	-0.46 (-1.85,0.93)
Observations	14,388	14,385	12,354	12,351	11,096	11,095	2,122
Num Patients	1,680	1,680	1,074	1,074	870	870	472

Propensity Score Weighting Metrics - Out of Control Cohorts

The area under the ROC curve and the standardized mean differences for the four cohorts are provided below.

<u>I. Hypertension: Age 18-59</u>

Logistic model for the Propensity Score

number of observations = 1680
area under ROC curve = 0.7158

Table 1: Patient characteristics of the cohorts in the Intervention vs Usual Care Clinic, before the first office visit, with propensity score weighting

	Intervention Clinic Mean	Usual Care Clinic Mean	p-value for diff.	Standardized diff.
prop_score	0.22	0.22	0.841	-0.014
Age	49.34	49.31	0.969	0.003
Female	0.42	0.43	0.638	-0.033
PtAsian	0.34	0.34	0.919	-0.007
PtHispanic	0.1	0.09	0.884	0.012
PtOther	0.16	0.17	0.886	-0.008
PtUnknown	0.04	0.04	0.997	0
Ins_HMO	0.19	0.19	0.966	-0.003
Ins_Medicaid	0.04	0.02	0.488	0.172
Ins_MedicareFFS	0.02	0.02	0.639	0.039
Ins_MedicareHMO	0	0	0.77	-0.014
Ins_Oth	0	0	.	.
PtCharlson	0.56	0.58	0.777	-0.016
Num_OV primary_care	3.13	3.27	0.482	-0.045
Num _OV_endocrinology	0.1	0.12	0.699	-0.024
Num _OV_nephrology	0.05	0.07	0.671	-0.015
Num _OV_cardiology	0.1	0.13	0.499	-0.038
Num _OV_allspecialist	3.45	3.42	0.932	0.005
Num _telephone_call	6.74	6.74	0.998	0

II. Hypertension: Age 60-80

Logistic model for the Propensity Score

number of observations = 1074
area under ROC curve = 0.7046

Table 2: Patient characteristics of the cohorts in the Intervention vs Usual Care Clinic, before the first office visit, with propensity score weighting

	Intervention Clinic Mean	Usual Care Clinic Mean	p-value for diff.	Standardized diff.
prop_score	0.14	0.14	0.846	0.013
Age	69.39	69.78	0.483	-0.065
Female	0.53	0.56	0.529	-0.064
PtAsian	0.28	0.26	0.627	0.05
PtHispanic	0.06	0.07	0.675	-0.03
PtOther	0.14	0.13	0.751	0.024
PtUnknown	0.03	0.03	0.921	-0.004
Ins_HMO	0.06	0.07	0.695	-0.03
Ins_Medicaid	0	0.01	0.013	-0.161
Ins_MedicareFFS	0.49	0.5	0.782	-0.028
Ins_MedicareHMO	0.13	0.13	1	0
Ins_Oth	0	0	.	.
PtCharlson	1.26	1.14	0.386	0.083
Num_OV primary_care	4.64	4.5	0.649	0.04
Num _OV_endocrinology	0.3	0.22	0.631	0.073
Num _OV_nephrology	0.23	0.22	0.924	0.009
Num _OV_cardiology	0.59	0.37	0.26	0.174
Num _OV_allspecialist	8.12	7.06	0.577	0.122
Num _telephone_call	11.17	10.41	0.632	0.069

III. Diabetes: Age 18-75

Logistic model for the Propensity Score

number of observations = 870
area under ROC curve = 0.7058

Table 3: Patient characteristics of the cohorts in the Intervention vs Usual Care Clinic, before the first office visit, with propensity score weighting

	**Intervention Clinic Mean**	**Usual Care Clinic Mean**	**p-value for diff.**	**Standardized diff.**
prop_score	0.30	0.31	0.502	-0.071
Age	60.12	59.28	0.372	0.078
Female	0.50	0.48	0.704	0.033
PtAsian	0.33	0.34	0.749	-0.025
PtHispanic	0.12	0.12	0.950	0.006
PtOther	0.18	0.19	0.948	-0.005
PtUnknown	0.04	0.05	0.556	-0.054
Ins_HMO	0.17	0.17	0.813	0.021
Ins_Medicaid	0.00	0.03	0.002	-0.278
Ins_MedicareFFS	0.29	0.26	0.419	0.078
Ins_MedicareHMO	0.05	0.06	0.718	-0.033
Ins_Oth	0.00	0.00	.	.
PtCharlson	2.28	2.15	0.478	0.094
Num_OV primary_care	4.75	4.71	0.909	0.011
Num _OV_endocrinology	0.58	0.50	0.654	0.050
Num _OV_nephrology	0.38	0.38	0.969	0.003
Num _OV_cardiology	0.36	0.33	0.740	0.023
Num _OV_allspecialist	7.69	7.42	0.768	0.024
Num _telephone_call	12.84	12.66	0.896	0.011

IV. Diabetes: A1c

Logistic model for the Propensity Score

number of observations = 472
area under ROC curve = 0.7295

Table 4: Patient characteristics of the cohorts in the Intervention vs Usual Care Clinic, before the first office visit, with propensity score weighting

	Intervention Clinic Mean	Usual Care Clinic Mean	p-value for diff.	Standardized diff.
prop_score	0.33	0.34	0.921	-0.015
Age	56.42	56.15	0.855	0.02
Female	0.51	0.48	0.57	0.068
PtAsian	0.32	0.33	0.877	-0.017
PtHispanic	0.17	0.18	0.886	-0.016
PtOther	0.16	0.16	0.883	-0.014
PtUnknown	0.04	0.04	0.898	0.014
Ins_HMO	0.2	0.19	0.79	0.029
Ins_Medicaid	0	0.02	0.008	-0.211
Ins_MedicareFFS	0.16	0.17	0.906	-0.012
Ins_MedicareHMO	0.1	0.08	0.628	0.111
Ins_Oth	0	0	.	.
PtCharlson	2.17	2.13	0.874	0.029
Num_OV primary_care	4.88	4.47	0.56	0.103
Num _OV_endocrinology	0.88	0.95	0.713	-0.039
Num _OV_nephrology	0.19	0.19	0.957	0.007
Num _OV_cardiology	0.33	0.34	0.931	-0.011
Num _OV_allspecialist	7.84	7.28	0.719	0.05
Num _telephone_call	14.69	12.78	0.563	0.127
