# Supplementary material for: Workflow standardization of a novel team care model to improve chronic care: a quasi-experimental study
Source: BMC Health Serv Res. 2017 Apr 19;17:286. doi: 10.1186/s12913-017-2240-1 (PMC5395876; doi:10.1186/s12913-017-2240-1)
Supplement: Supplementary file 4 — Adjusted A1c Outcomes for Diabetes Patients with Pre-visit A1c ≥ 9.0, A1c ≥ 8.5, and A1c ≥ 8.0. This file includes the results of Table 5 for diabetes patients with Pre-visit A1c ≥ 9.0, A1c ≥ 8.5, and A1c ≥ 8.0. (PDF 313 kb) [file 12913_2017_2240_MOESM4_ESM.pdf]

Additional File 4: Adjusted A1c Outcomes for Diabetes Patients with Pre-visit A1c ≥ 9.0, A1c ≥ 8.5, and A1c ≥ 8.0

Table 5. Adjusted Results from a Piecewise Linear Growth Curve Model of A1c for Diabetes Patients with a Primary Care Office Visit – Pre-visit A1c ≥ 9.0, A1c ≥ 8.5, and A1c ≥ 8.0

	**Diabetes** ***A1c ≥ 9.0*** Est. (95% CI)	**Diabetes** ***A1c ≥ 8.5*** Est. (95% CI)	**Diabetes** ***A1c ≥ 8.0*** Est. (95% CI)
Intervention Clinic	-4.6 (-14.80,5.61)	-3.33 (-11.41,4.75)	0.52 (-1.26,2.31)
Before First Office Visit	0.63 (0.35,0.91)**	0.5 (0.28,0.72)**	0.47 (0.29,0.65)**
1-180 Days Post Visit	-1.83 (-2.27,-1.38)**	-1.18 (-1.55,-0.82)**	-0.83 (-1.13,-0.54)**
181-365 Days Post Visit	1 (0.17,1.84)*	0.35 (-0.30,1.00)	0.23 (-0.27,0.73)
Intervention Clinic x Before Visit	0.07 (-0.16,0.30)	0.09 (-0.07,0.25)	0.07 (-0.05,0.19)
Intervention Clinic x 1-180 Days Post Visit	-0.66 (-1.33,0.003)	-0.71 (-1.21,-0.21)**	-0.75 (-1.14,-0.36)**
Intervention Clinic x 181-360 Days Post Visit	-0.46 (-1.85,0.93)	-0.08 (-1.24,1.09)	0.19 (-0.84,1.23)
Observations	2,122	3,311	4,989
Num Patients	472	692	978
